# Characteristics and therapeutic outcomes of subcutaneous panniculitis-like T-cell lymphoma with and without germline *HAVCR2* mutations in Thai children and adolescents

**DOI:** 10.1186/s13023-024-03438-w

**Published:** 2024-11-13

**Authors:** Pimpitcha Youthong, Samart Pakakasama, Patcharee Komvilaisak, Piya Rujkijyanont, Chane Choed-Amphai, Kamon Phuakpet, Chatphatai Moonla, Chantana Polprasert, Darintr Sosothikul

**Affiliations:** 1 Department of Pediatrics, Faculty of Medicine, Chulalongkorn University and King Chulalongkorn Memorial Hospital, Bangkok, Thailand; 2 Department of Pediatrics, Faculty of Medicine Ramathibodi Hospital, Mahidol University, Bangkok, Thailand; 3 Department of Pediatrics, Faculty of Medicine, Khon Kaen University, Khon Kaen, Thailand; 4 Department of Pediatrics, Phramongkutklao Hospital and Phramongkutklao College of Medicine, Bangkok, Thailand; 5 Department of Pediatrics, Faculty of Medicine, Chiang Mai University, Chiang Mai, Thailand; 6 Department of Pediatrics, Faculty of Medicine Siriraj Hospital, Mahidol University, Bangkok, Thailand; 7 Department of Medicine, Faculty of Medicine, Chulalongkorn University and King Chulalongkorn Memorial Hospital, Bangkok, Thailand; 8 Center of Excellence in Translational Hematology, Faculty of Medicine, Chulalongkorn University, Bangkok, Thailand; 9 Integrative and Innovative Hematology/Oncology Research Unit, Department of Pediatrics, Faculty of Medicine, Chulalongkorn University, Bangkok, Thailand

**Keywords:** Children, *HAVCR2*, Hemophagocytic lymphohistiocytosis, Immunosuppressive agent, Subcutaneous panniculitis-like T-cell lymphoma

## Abstract

**Background:**

Subcutaneous panniculitis-like T-cell lymphoma (SPTCL) is a rare subtype of non-Hodgkin lymphoma associated with hemophagocytic lymphohistiocytosis (HLH)/HLH-like systemic illnesses and germline *HAVCR2* mutations. Although previous studies suggested successful treatment of SPTCL with immunosuppressive therapy (IST) without chemotherapy, IST data in pediatric SPTCL remain scarce. To explore characteristics and therapeutic outcomes, comparing IST-based and chemotherapy-based regimens in pediatric SPTCL, in this retrospective cohort study, patients with SPTCL diagnosed at age ≤20 years during 2007-2023 were enrolled from 6 hematology/oncology centers in Thailand. *HAVCR2* exon 2 sequencing was performed using DNA extracted from peripheral blood or bone marrow. Presence of HLH/HLH-like systemic illnesses, treatment outcomes, and adverse events (AEs) were reviewed and analyzed.

**Results:**

Of 22 patients with SPTCL (median age at diagnosis, 11.5 years [range, 6.0-19.0]; 63.6% males), 86.4% harbored germline *HAVCR2* mutation, either homozygous (77.3%) or heterozygous (9.1%) p.Y82C variant, while 68.2% developed HLH/HLH-like systemic illnesses. Overall, 36.4% received IST as first-line treatment. Durable complete remission (CR) was achieved in 71.4% and 50.0% after first-line chemotherapy and IST, respectively (*P*=0.45); however, chemotherapy tended to increase any AEs compared to IST (57.1% vs. 12.5%; *P*=0.07). The most common AEs were hypertension (27.3%), febrile neutropenia (18.2%), and fungal infection (13.6%). Among the relapsed cases, 71.4% could reach CR after subsequent-line therapy. Fatality (4.5%) only occurred in the chemotherapy group.

**Conclusions:**

Pediatric SPTCL in Thailand frequently involves germline *HAVCR2* mutations and/or HLH/HLH-like systemic illnesses. With comparable response and modest therapy-related toxicity, IST-based regimens may alternatively be considered as first-line treatment for pediatric SPTCL.

## Introduction

Subcutaneous panniculitis-like T-cell lymphoma (SPTCL) is a distinctive subtype of T-cell non-Hodgkin lymphoma (NHL), accounting for <1% of all NHL cases worldwide [1]. Despite its rarity, SPTCL can affect both male and female individuals in all age groups, with the classical manifestation of subcutaneous nodules infiltrated by atypical lymphoid cells rimming adipocytes with positive immunohistochemistry for CD3, CD8, T-cell intracytoplasmic antigen-1 (TIA-1) and T-cell receptor beta F1 (BF1) [2]. However, in addition to localized lesions, SPTCL has a strong association with hemophagocytic lymphohistiocytosis (HLH), a life-threatening condition accompanied by devastating immune activation and cytokine release [1–4]. To elucidate the pathogenesis of SPTCL/HLH clinical spectrum, germline mutations in the hepatitis A virus cellular receptor (*HAVCR2*) gene have been discovered to alter the T-cell immunoglobin and mucin-domain containing-3 (TIM-3) protein leading to immune dysregulation and amplified inflammatory signaling [5, 6]. A recent individual patient data (IPD) level meta-analysis not only confirmed an increased risk of HLH/HLH-like systemic illnesses among patients with homozygous and heterozygous *HAVCR2* mutations, but also suggested an age-dependent tendency to develop HLH/HLH-like systemic illnesses in children and young adults with SPTCL rather than in elderly patients [7].

As SPTCL is classified as one of peripheral T-cell NHL, multidrug chemotherapy (CMT)-based regimens have traditionally been considered as a specific treatment for SPTCL, with reported complete response (CR) rates ranging from 28.5% to 80.0% [4, 8–11]. However, based on the shared pathogenesis between SPTCL and HLH in the presence of *HAVCR2* mutations, immunosuppressive therapy (IST), which is a mainstay treatment for HLH [12], holds potential as a therapeutic approach for patients with SPTCL, especially in younger patients who frequently harbor *HAVCR2* mutations and manifest with HLH/HLH-like systemic illnesses [7]. Previous retrospective cohorts demonstrated that although both CMT-based and IST-based regimens can effectively induce CR for SPTCL [4, 8–11], adverse events (AEs) tend to occur among those receiving CMT [11]. Nevertheless, studies to directly determine the efficacy and safety of IST-based compared with CMT-based regimens in SPTCL, particularly in childhood and adolescence, are scarce [11].

To address this knowledge gap, we conducted a multicenter retrospective cohort study involving Thai children and adolescents diagnosed with SPTCL to comprehensively examine its clinical characteristics, presence of germline *HAVCR2* mutations, occurrence of HLH/HLH-like systemic illnesses, therapeutic regimens, disease outcomes and post-treatment AEs. Comparisons of all outcomes of interest were prespecified based on groups of initial treatment, IST-based vs. CMT-based regimens, for pediatric SPTCL.

## Methods

### Study participants and study design

This multicenter retrospective cohort study enrolled Thai patients who were diagnosed with SPTCL at age ≤20 years between 2007 and 2023 from 6 hematology/oncology centers. Four centers are located in the central region (Faculty of Medicine, Chulalongkorn University and King Chulalongkorn Memorial Hospital; Faculty of Medicine Ramathibodi Hospital, Mahidol University; Faculty of Medicine Siriraj Hospital, Mahidol University; and Phramongkutklao Hospital, Phramongkutklao College of Medicine), whereas 1 center is in the northern region (Faculty of Medicine, Chiang Mai University) and 1 center is in the northeastern region (Faculty of Medicine, Khon Kaen University) of Thailand. The study protocol was ethically approved by the Institutional Review Boards of all respective study sites. Written informed consent forms were obtained from each patient and/or their parents/guardians prior to participation in the study, in accordance with the Declaration of Helsinki.

The pathological diagnosis of SPTCL was verified by hematopathologists at the study sites based on immunohistochemistry and T-cell receptor gene rearrangement studies, as previously described [6]. HLH was defined by the HLH-2004 criteria [13], while partially met criteria (fever, cytopenias, serum ferritin ≥500 µg/L, and presence of hemophagocytosis in bone marrow) were used to define HLH-like systemic illnesses [7]. The medical records were retrospectively reviewed for clinical data, focusing on the treatments and outcomes of interest. Since a standard treatment approach did not exist at the time of this study conducted, therapeutic regimens for SPTCL with or without HLH/HLH-like systemic illnesses were based on the discretion of the treating physicians. IST-based regimens were any regimens of corticosteroids and/or immunomodulatory drugs without any chemotherapeutic agents, whereas regimens containing ≥1 chemotherapeutic agents were classified as CMT-based regimens regardless of the concomitant use of corticosteroids. The primary outcome was the CR rates after first-line treatment without relapse. The number of patients with any AEs and the CR rates at the last follow-up were secondary outcomes. The disease response was evaluated using the revised International Working Group (IWG) response criteria and definitions [14].

Peripheral blood or bone marrow aspirates from all subjects were transferred to the central laboratory (Center of Excellence in Translational Hematology, Faculty of Medicine, Chulalongkorn University) for the detection of germline *HAVCR2* mutations, as previously described [6, 7]. Briefly, DNA extraction was performed using Gentra Puregene^®^ Blood Kits (QIAGEN N.V., Hilden, Germany). The primers to detect *HAVCR2* exon 2 mutations included forward primer (5’-GGAAGCTGAGGGTGTATTTCT-3’) and reverse primer (5’-TCAGAGCCAGCTAAAGATTCC-3’), which could cover all 3 pathogenic variants reported in the literature (p.Y82C, p.I97M and p.T101I). The polymerase chain reaction products were then purified and sent for Sanger sequencing (Macrogen Inc., Seoul, South Korea).

### Statistical analysis

The Statistical Product and Service Solutions (SPSS) software version 29.0 (SPSS Inc., Chicago, IL) was employed for all statistical analyses. In descriptive statistics, categorical data between groups receiving IST-based or CMT-based regimens were compared using the chi-square test or Fisher’s exact test, while continuous data were compared using the nonparametric Wilcoxon rank-sum test since the small sample size was anticipated due to the extremely low prevalence of SPTCL. The statistical significance was considered when *P*<0.05.

## Results

### Patient characteristics

A total of 22 patients with SPTCL were enrolled during the study period. The median age at the time of diagnosis was 11.5 years (range, 6.0 to 19.0) with a male sex predominance (63.6%). All patients had multiple skin lesions, usually in the trunk (59.1%), upper extremities (63.6%), and lower extremities (59.1%); the most common form was subcutaneous nodules (86.4%). Overall, there were 15 patients (68.2%) who developed HLH/HLH-like systemic illnesses; 14 patients fully met the HLH-2004 criteria for HLH, while the other patient partially met the criteria as HLH-like systemic illnesses. Hyperferritinemia (≥500 µg/L; 77.3%) was the abnormal laboratory finding most frequently observed, followed by anemia (72.7%), hypertriglyceridemia (≥265 mg/dL; 63.6%), neutropenia (50.0%), hypofibrinogenemia (≤1.5 g/L; 27.3%), and thrombocytopenia (<100 x 10^9^ platelets/L; 18.2%). Analysis of the *HAVCR2* gene revealed germline homozygous *HAVCR2* p.Y82C mutation in 17 patients (77.3%), heterozygous *HAVCR2* p.Y82C mutation in 2 patients (9.1%), and *HAVCR2* wild-type in 3 patients (13.6%). None of the patients were identified with *HAVCR2* p.I97M or p.T101I variants. Notably, of the 19 patients with *HAVCR2* p.Y82C variant, 3 homozygotes (15.8%) had an episode of idiopathic HLH/HLH-like systemic illnesses before the appearance of panniculitis-like skin lesions with recurrent HLH/HLH-like systemic illnesses.

Based on the Genome Aggregation Database (gnomAD), although *HAVCR2* p.Y82C mutation in the general population is rare with a minor allele frequency (MAF) of 3.6 x 10^-3^, its ethnicity-specific MAFs are variable, ranging from 4.2 x 10^-5^ in Africans to 2.1 x 10^-2^ in East Asians [5, 6]. In Southeast Asia, where *HAVCR2* p.Y82C MAF is 1.6 x 10^-3^ [5, 6], this pediatric cohort in which 86.4% of the patients were *HAVCR2* p.Y82C homozygotes or heterozygotes confirmed an increased risk of developing SPTCL in the presence of *HAVCR2* p.Y82C allele (odds ratio, 2.9 x 10^3^; 95% confidence interval, 1.3 x 10^3^ to 6.1 x 10^3^; *P*<0.0001; for MAF), corresponding to that previously reported [6].

According to first-line treatment administered to the patients with SPTCL with or without HLH/HLH-like systemic illnesses, 8 patients initially received IST-based regimens including prednisolone monotherapy (n=2), prednisolone plus cyclosporine (n=4), and dexamethasone plus cyclosporine and intravenous immunoglobulin (n=2). On the contrary, in the other 14 patients, CMT-based regimens, including the HLH-94 protocol containing etoposide (n=3), CHOP (cyclophosphamide, doxorubicin, vincristine, and prednisolone) or CHOP-like regimens (n=3), and other protocols for high-risk anaplastic large cell lymphoma [15] or peripheral T-cell lymphoma (n=8), were implemented. The baseline characteristics of the 22 included patients with SPTCL are outlined in detail in Table 1 and Table 2.

**Table. 1:** Summary of baseline characteristics of 22 patients with SPTCL included in the study

Characteristics	Statistical value
Male sex (n, %)	14 (63.6)
Median age at diagnosis of SPTCL (y, range)	11.5 (6.0–19.0)
Past history of HLH/HLH-like systemic illnesses	3 (13.6)
*Clinical manifestation at diagnosis of SPTCL*	
Presence of HLH/HLH-like systemic illnesses	15 (68.2)
Skin lesion	22 (100)
Fever	21 (95.5)
Hepatomegaly	13 (59.1)
Splenomegaly	13 (59.1)
Lymphadenopathy	4 (18.2)
Neck or submandibular mass	3 (13.6)
Periorbital swelling	3 (13.6)
Weight loss	3 (13.6)
Parotid gland enlargement	1 (4.5)
*Morphology of skin lesion*	
Subcutaneous nodule	19 (86.4)
Patch or plaque	5 (22.7)
Macule or papule	2 (9.1)
Ulceration	1 (4.5)
>1 morphology	5 (22.7)
*Localization of skin lesion*	
Head or face	5 (22.7)
Trunk	13 (59.1)
Upper extremities	14 (63.6)
Lower extremities	13 (59.1)
*HAVCR2 mutational status*	
Wild-type	3 (13.6)
Homozygous *HAVCR2*^Y82C^	17 (77.3)
Heterozygous *HAVCR2*^Y82C^	2 (9.1)
*First-line treatment*	
IST-based	8 (36.4)
Chemotherapy-based	14 (63.6)

*HLH* hemophagocytic lymphohistiocytosis, *IST* immunosuppressive therapy, *SPTCL* subcutaneous panniculitis-like T-cell lymphoma

**Table. 2:** Clinical manifestation and treatments of 22 patients with SPTCL included in the study

No.	Sex	Age at diagnosis (y)	Prior HLH	Site(s) of skin lesion	HLH/HLH-like systemic illnesses	Fever	LN	Hepatomegaly/splenomegaly	Cytopenia	*HAVCR2* mutation	First-line treatment	Relapsed disease after first-line treatment	Subsequent-line treatment	Disease status at the last follow-up
1	F	13	Yes	T, L	Present	Yes	No	No	Yes	Homozygous	IST	No	No	CR
2	M	14	No	U, L	Present	Yes	Yes	Yes	Yes	Homozygous	IST	No	No	CR
3	M	12	No	T	Absent	Yes	No	No	Yes	Homozygous	IST	No	No	CR
4	F	8	No	H, T, U, L	Present	Yes	No	Yes	Yes	Homozygous	IST	Yes	CMT	CR
5	M	10	No	T, U, L	Present	Yes	Yes	Yes	Yes	Homozygous	IST	Yes	CMT	CR
6	M	6	No	H, U, L	Absent	Yes	No	Yes	Yes	Homozygous	IST	No	No	CR
7	F	19	No	U	Present	Yes	No	Yes	Yes	Heterozygous	IST	Yes	IST	CR
8	F	11	No	T, U, L	Absent	Yes	No	No	N/A	Heterozygous	IST	Unknown	No	Unknown
9	M	9	No	T	Present	Yes	No	Yes	Yes	Homozygous	CMT	No	No	CR
10	F	13	No	U, L	Present	Yes	No	No	Yes	Homozygous	CMT	No	No	CR
11	M	9	Yes	T, U, L	Present	Yes	Yes	Yes	Yes	Homozygous	CMT	No	No	CR
12	M	14	No	T, U	Present	Yes	No	Yes	No	Homozygous	CMT	No	No	CR
13	M	10	No	T, U	Present	Yes	No	Yes	Yes	Homozygous	CMT	Yes	IST	Dead
14	M	11	No	T, L	Absent	Yes	No	No	Yes	Homozygous	CMT	Yes	CMT	CR
15	F	8	No	L	Absent	Yes	No	Yes	Yes	Homozygous	CMT	Yes	IST	CR
16	M	9	No	H (eyelid)	Present	Yes	No	Yes	Yes	Homozygous	CMT	No	No	CR
17	M	15	Yes	U, L	Present	Yes	No	Yes	No	Homozygous	CMT	Yes	CMT	Unknown
18	M	9	No	T, L	Present	Yes	No	Yes	Yes	Homozygous	CMT	No	No	CR
19	M	12	No	T, U	Absent	Yes	Yes	Yes	No	Homozygous	CMT	No	No	CR
20	F	15	No	H, U	Absent	No	No	No	Yes	Wild-type	CMT	No	No	CR
21	F	16	No	T, U, L	Present	Yes	No	No	Yes	Wild-type	CMT	No	No	CR
22	M	14	No	H (eyelid)	Present	Yes	No	Yes	Yes	Wild-type	CMT	No	No	CR

*CMT* chemotherapy, *CR* complete remission, *F* female, *H* head, *HLH* hemophagocytic lymphohistiocytosis, *IST* immunosuppressive therapy, *L* lower extremity, *LN* lymphadenopathy, *M* male, *N/A* not available, *T* trunk, *U* upper extremity

### Disease response to IST-based vs. CMT-based regimens

After first-line IST (n=8) or CMT (n=14), durable CR without relapse was achieved in 4 patients (50.0%) of the IST group and 10 patients (71.4%) of the CMT group (*P*=0.45), although 1 patient (4.5%) in the IST group was lost to follow-up before complete treatment. There were no statistical differences in the presence of HLH/HLH-like systemic illnesses between the groups (62.5% in the IST group vs. 71.4% in the CMT group; *P*=0.67). Nonetheless, all the patients receiving IST-based regimens (100%) harbored homozygous or heterozygous *HAVCR2* p.Y82C mutations while 78.6% of those receiving CMT-based regimens had homozygous *HAVCR2* p.Y82C mutation (*P*=0.10). None of the patients with *HAVCR2* wild-type (n=3) received IST-based regimens in this study. In the subgroup analysis of patients with *HAVCR2* p.Y82C mutations (n=19), the CR rates without relapse after first-line treatment using IST-based or CMT-based regimens were similar (*P*=0.47).

Among 7 cases with relapsed disease after first-line treatments (31.8%), 3 patients (42.8%) were treated with IST-based regimens, including cyclosporine monotherapy (n=2) and prednisolone plus cyclosporine (n=1), as subsequent-line treatment. While the other 4 patients received CMT-based regimens, including the HLH-94 protocol containing etoposide (n=1), CHOP-like regimen (n=1), and other protocols for peripheral T-cell lymphoma (n=2). One patient who was treated with a CMT-based regimen for relapsed disease had undergone autologous hematopoietic stem cell transplant but was subsequently lost to follow-up. Overall, CR in cases with relapsed disease could be induced by subsequent-line IST-based and CMT-based regimens in 2 of 3 patients (66.7%) and 3 of 4 patients (75.0%), respectively. Until the last follow-up, the CR rates among the patients receiving IST-based regimens (87.5%) and CMT-based regimens (85.7%) as first-line therapy were not statistically different (*P*=0.69). The overall CR rate in this study was 86.4% during the median follow-up time of 20.0 months (range, 1.5 to 194.6). Comparisons of disease response after first-line treatments, after subsequent-line treatments for relapsed disease, and at the last follow-up, between the IST and CMT groups are shown in Table 3.

**Table. 3:** Outcome comparisons between groups receiving IST-based or CMT-based regimens as a first-line treatment for SPTCL

Characteristics or outcomes of interest	IST-based (N=8)	CMT-based (N=14)	*P*-value
*Sex (n, %)*			0.39
Male	4 (50.0)	10 (71.4)	
Female	4 (50.0)	4 (28.6)	
Median age at diagnosis of SPTCL (y, range)	11.5 (6.0–19.0)	10.0 (8.0–16.0)	0.93
Presence of HLH/HLH-like systemic illnesses	5 (62.5)	10 (71.4)	0.67
*HAVCR2* *mutational status*			0.10
Wild-type	0 (0)	3 (21.4)	
Homozygous *HAVCR2*^Y82C^	6 (75.0)	11 (78.6)	
Heterozygous *HAVCR2*^Y82C^	2 (25.0)	0 (0)	
*Disease status after first-line treatment*			0.45
Complete remission without relapse	4 (50.0)	10 (71.4)	
Relapsed disease	3 (37.5)	4 (28.6)	
Unknown status*	1 (12.5)	0 (0)	
*Disease status after subsequent-line treatment in relapsed cases (n=7)*			0.64
Complete remission	3 (37.5)	2 (14.3)	
Complete remission by IST	1 (12.5)	1 (7.1)	
Complete remission by CMT	2 (25.0)	1 (7.1)	
Dead	0 (0)	1 (7.1)	
Unknown status*	0 (0)	1 (7.1)	
*Disease status at the last follow-up*			0.69
Complete remission	7 (87.5)	12 (85.7)	
Dead	0 (0)	1 (7.1)	
Unknown status*	1 (12.5)	1 (7.1)	
*Adverse events after treatment*			
Any adverse events	1 (12.5)	8 (57.1)	0.07
Hypertension	1 (12.5)	5 (35.7)	0.35
Febrile neutropenia	1 (12.5)	3 (21.4)	0.60
Fungal infection	1 (12.5)	2 (14.3)	0.91
Acute kidney injury	0 (0)	2 (14.3)	0.52
Transaminitis	0 (0)	2 (14.3)	0.52
Septic shock	0 (0)	1 (7.1)	0.44
Dead	0 (0)	1 (7.1)	0.44

*CMT* chemotherapy, *HLH* hemophagocytic lymphohistiocytosis, *IST* immunosuppressive therapy, *SPTCL* subcutaneous panniculitis-like T-cell lymphoma

^*^Unknown status due to loss to follow-up before complete treatment

### Treatment-related complications

Any treatment-related AEs were observed in only 1 of 8 patients in the IST group (12.5%), but in 8 of 14 patients (57.1%) in the CMT group which included a fatal complication (*P*=0.07). Common AEs were hypertension (27.3%), febrile neutropenia (18.2%), and fungal infection (13.6%). The other AEs that occurred less frequently were acute kidney injury (14.3%), transaminitis (14.3%), and sepsis with septic shock (7.1%). Although serious infections were rare, a patient in the CMT group who developed septic shock due to bacterial sepsis and disseminated fungal infection had progressed to multiorgan failure and disseminated intravascular coagulation and eventually succumbed to these complications. The mortality rate is 4.5% for the whole cohort. The numbers of patients with AEs in the IST group and the CMT group are described in Table 3.

## Discussion

In this multicenter study that collected data from 22 Thai children and adolescents diagnosed with SPTCL over the past 16 years, we discovered that most of the cases harbored germline *HAVCR2* mutations (86.4%), either homozygous or heterozygous, and manifested with HLH/HLH-like systemic illnesses (68.1%) at the time of SPTCL diagnosis. Compared to the prevalence of *HAVCR2* mutations (24.5% to 84.6%) and HLH/HLH-like systemic illnesses (17.0% to 63.0%) reported in previous studies among patients with SPTCL of all age groups [5–7, 16, 17], SPTCL in the Thai pediatric population showed a higher burden of underlying *HAVCR2* mutations as a predisposing factor for developing SPTCL associated with HLH/HLH-like systemic illnesses in the young. Furthermore, with the excellent prognosis of the disease, our study demonstrated that initial treatment with IST-based or CMT-based regimens was comparably effective to induce CR without relapse, while the treatment-related AEs tended to increase among those in the CMT group although statistical significance was not reached. These findings on the disease response to IST-based regimens in pediatric SPTCL align with the results of a previous study [11], supporting that IST-based regimens can be a valuable first-line treatment for SPTCL in children and adolescents, regardless of the presence of HLH/HLH-like systemic illnesses, to avoid toxicity of chemotherapeutic agents.

The misfolding of the TIM-3 protein caused by germline *HAVCR2* mutations is responsible for persistent immune activation in the pathogenesis of SPTCL/HLH [5]. Not only does it increase the risk of developing HLH/HLH-like systemic illnesses in patients with SPTCL, but the high prevalence of *HAVCR2* mutations in our pediatric SPTCL population suggests that it can also accelerate the development of the disease at a young age. In contrast, as previously studied in Thailand, *HAVCR2* mutations were less prevalent among patients diagnosed with SPTCL at age >20 years (62.5% to 83.3%) [6, 7]. This constellation is consistent with the results of a recent IPD level meta-analysis that patients with *HAVCR2* mutations, both homozygous and heterozygous, manifested with SPTCL with or without HLH/HLH-like systemic illnesses earlier than those with *HAVCR2* wild-type [7]. Apart from the age-dependent SPTCL/HLH clinical spectrum, *HAVCR2* mutations can contribute to HLH/HLH-like systemic illnesses alone, or idiopathic HLH/HLH-like systemic illnesses, without evidence of SPTCL or preceding SPTCL [7, 18, 19]. Our findings that the patients with germline homozygous *HAVCR2* mutation were able to present with idiopathic HLH/HLH-like systemic illnesses before the onset of SPTCL help confirm this hypothesis. Additionally, based on *HAVCR2* mutation-associated immune dysregulation, immunosuppressive or immunomodulatory agents should directly counteract the disease mechanism [5]. Hence, since pediatric SPTCL increases the likelihood of harboring *HAVCR2* mutations, the use of IST-based regimens in this setting of patients would be an appropriate therapeutic approach instead of aggressive CMT-based regimens. In clinical practice, the detection of *HAVCR2* mutations in children and adolescents with SPTCL with or without HLH/HLH-like systemic illnesses, and in patients with idiopathic HLH/HLH-like systemic illnesses, should be helpful in guiding treatment selection and predicting the natural history of the disease.

The ultimate objective of our study is to compare the differences in disease response and treatment-related AEs between groups receiving IST-based or CMT-based regimens as first-line therapy. Our study indicated that the CR rate without relapse of the CMT group was not significantly superior to that of the IST group. Nevertheless, CMT-based regimens led to a tendency to increase any AEs, including febrile neutropenia, infectious complications, acute kidney injury, transaminitis, and hypertension, compared to IST-based regimens. As the major AE of multidrug CMT is bone marrow suppression leading to an increased risk of neutropenia and serious infections, the consideration of IST as an alternative treatment with equivalent efficacy but potentially less toxicity for SPTCL, especially in the pediatric population, is ethically reasonable. In this study, since all the patients with *HAVCR2* wild-type were treated with CMT-based regimens and most patients had *HAVCR2* mutations, the interpretation of the benefits of IST-based regimens in relation to *HAVCR2* mutational status would be limited. However, several studies in SPTCL of all age groups previously showed that IST-based regimens can induce an excellent disease response in both *HAVCR2*-mutated and *HAVCR2* wild-type groups [7, 16]. Therefore, based on current evidence, IST-based regimens should be a preferred first-line treatment for patients with SPTCL in all age groups, especially in the presence of *HAVCR2* mutations, although specific guidelines for SPTCL have not been established [20].

Our study has several limitations. First, although this study is the largest among the pediatric SPTCL population in Southeast Asia, the rarity of the disease led to a limited number of study participants, making the statistical power less sufficient to detect outcome differences between groups receiving IST-based or CMT-based regimens. Second, the retrospective study design would cause information bias, especially in patients who were lost to follow-up. Third, there were variations in CMT-based regimens between the study sites, limiting the interpretation of regimen-specific effects in the CMT group. Fourth, the fraction of patients with *HAVCR2* wild-type was small; therefore, outcome comparisons between groups with or without *HAVCR2* mutations could not be performed. The influence of *HAVCR2* mutations on an increased risk of relapse, as shown in the other East Asian SPTCL population [17], was not analyzable. Fifth, only *HAVCR2* p.Y82C variant was identified in our study population of exclusively Thai pediatric patients with SPTCL. Due to the variations in *HAVCR2* p.Y82C MAFs between different ethnicities that would affect its allele frequencies among patients with SPTCL [5, 6], our findings might be less generalizable to SPTCL populations other than Southeast Asians. Larger well-designed case-control studies, e.g., IST-treated vs. CMT-treated and *HAVCR2*-mutated vs. *HAVCR2* wild-type, which include various ethnic groups of patients with SPTCL, deserve further investigation to enhance the statistical power and generalizability of the results.

## Conclusions

The prevalence of germline *HAVCR2* mutations and HLH/HLH-like systemic illnesses is high among Thai children and adolescents with SPTCL. Despite the limited interpretation due to the small sample size, initiating SPTCL-specific treatment with IST-based regimens is as effective as CMT-based regimens in inducing CR without relapse, with a nonsignificant tendency to minimize the regimen-related toxicity. Regardless of the presence of HLH/HLH-like systemic illnesses, IST-based regimens could alternatively be considered as first-line therapy for pediatric patients with SPTCL, particularly those with *HAVCR2* mutations. Future multicenter studies across various ethnicities with larger numbers of patients are warranted to provide more robust findings on the efficacy and safety of these regimens in pediatric SPTCL.

## Data Availability

The data that support the findings of this study are not openly available due to reasons of sensitivity and are available from the corresponding author upon reasonable request.
